# Promoting User Involvement to Foster Technological Citizenship in the Digitizing Healthcare Domain

**DOI:** 10.1007/s11948-025-00565-w

**Published:** 2025-11-20

**Authors:** Anne Marte Gardenier, Iris Cramer, Rinie van Est

**Affiliations:** 1 Department of Industrial Engineering and Innovation Sciences, Technology Innovation and Society, Eindhoven University of Technology, Groene Loper 3, Eindhoven, 5612 AE The Netherlands; 2 Department of Electrical Engineering, Eindhoven University of Technology, Groene Loper 3, Eindhoven, 5612 AE The Netherlands; 3 Department of Anesthesiology, Intensive Care and Pain Medicine, Catharina Hospital, Eindhoven, The Netherlands; 4 Rathenau Instituut, Anna van Saksenlaan 51, The Hague, 2593 HW The Netherlands

**Keywords:** Health care technology, Artificial intelligence, Technological citizenship, Digitization, User involvement, Socio-technical system, Continuous monitoring camera

## Abstract

Artificial intelligence (AI) is playing an increasingly prominent role in healthcare technology, particularly in patient monitoring and diagnosis. While AI offers significant benefits, it raises concerns about the patient-provider relationship and key care values. To mitigate these risks and align technology with societal values, experts stress the importance of user involvement in technology development. However, input from patients or nurses during AI development remains uncommon. The rapidly digitizing healthcare thus demonstrates a context where technological citizenship is not yet thriving, underscoring the need for improvement in this area. This article examines a case study where nurses contributed to the development of an AI-driven monitoring camera for the cardiothoracic surgery department. Based on interview data, our study shows that involving users, particularly nurses, can lead to the improved innovation of a socio-technical care practice, greater recognition of the importance of user involvement among technology developers, and increased empowerment of nurses with regards to the technology that impacts their work practice. To promote technological citizenship in the evolving healthcare landscape, this article offers recommendations for fostering greater user involvement during the development and implementation of new technologies.

**Supplementary Information:**

The online version contains supplementary material available at 10.1007/s11948-025-00565-w.

## Introduction

Artificial intelligence (AI) is playing an increasingly prominent role in healthcare technology, particularly in patient monitoring and diagnosis. One of its emerging applications is the continuous monitoring of patients. While this offers significant opportunities, it also raises ethical concerns. Even though patients in hospital wards are already continuously observed by a combination of nurses, doctors, and existing technologies, the introduction of AI disrupts the current system of human checks and balances.

One of the ethical risks lies in AI’s potentially deskilling effect on health care professionals’ ability to diagnose and conduct physical examination. This raises concerns that go beyond the direct clinical practice and touch on deeper normative issues, including professional integrity and public trust (Natali et al., 2025). Indeed, the use of AI in healthcare affects not only individuals directly involved in care but also has broader societal implications for institutions and society (Smallman, 2022).

The use of AI also impacts the relationship between healthcare professionals and patients (Steering Committee for Human Rights et al., 2024). When patients expect personal attention but are instead monitored by a computer-controlled camera, this mismatch can lead to stress and uncertainty—factors that may hinder recovery.

To address such ethical risks and to ensure that technological applications align with societal values, authors have argued that involving users during the development process is crucial (e.g., Boshuijzen-van Burken et al., 2023; Friedman, 1996; Kensing & Greenbaum, 2013; Schot & Rip, 1997; van der Velden & Mörtberg, 2015). Specifically with regard to technology for healthcare, authors have demonstrated that user involvement is essential to ensure alignment with user needs and values (Cenci et al., 2023; Walker et al., 2020; Weßel et al., 2022).

In practice, however, user involvement, for instance by patients or nurses, during the development of AI for healthcare is infrequent (Adus et al., 2023; Seibert et al., 2023; Vincenzi et al., 2024; von Gerich et al., 2022). New forms of interdisciplinary collaboration are emerging, but the process is often slow and not proportional to the rapid pace of technological advancement (Zidaru et al., 2021). Consequently, there is an expanding body of literature advocating for increased user involvement, including lay users, nurses, and patients, in the development of AI for healthcare to ensure risks can be averted (Lambert et al., 2023; O’Connor et al., 2023; Zhou et al., 2021).

This indicates that the institutions of science and healthcare currently insufficiently support users’ involvement in the technology development process. The rapidly digitizing healthcare sector highlights a context where technological citizenship is not yet thriving. Technological citizenship refers to citizens or lay users’ ability to navigate the opportunities and risks of digitization, contributing to the development and governance of digital technologies in society (Gardenier et al., 2024). The fact that stakeholders beyond developers, such as nurses, rarely have a say in the development of healthcare technology underscores the need to improve technological citizenship in healthcare. This is particularly relevant given the global shortage of nurses (Drennan & Ross, 2019) and the continuous digitization affecting their work. Therefore, this article addresses the question: How can technological citizenship be promoted in the digitizing healthcare domain?

This article answers this question through a case study analysis supplemented by interviews. In this case study, nurses, as users, were actively involved in the development process of an AI-driven monitoring camera for the cardiothoracic surgery department of the Catharina Hospital in The Netherlands. In this project, the different interests of developers and nurses emerged and influenced each other throughout the research process. The development process was a dynamic process where these interests evolved and interacted over time (Binder et al., 2015). In this article, we demonstrate the effects of this involvement on the care practice, the team of technology developers, and the nurses themselves. From this case, we extract lessons for promoting technological citizenship in the healthcare domain, guided by two research questions:

1. What are the results of involving nurses in the development of AI-driven healthcare technology?
2. What is the significance of involving nurses in the development of AI-driven healthcare technology?

Our study shows that involving nurses can lead to the improved innovation of a socio-technical care practice, greater recognition of the importance of user involvement among technology developers, and increased empowerment of nurses with regards to the technology that impacts their work practice. Therefore, to promote technological citizenship in the digitizing healthcare domain, this article demonstrates that it is essential to facilitate user involvement during the development and implementation of new technology. To promote this goal, we present recommendations to further support the involvement of nurses in this process. While this study focuses on enhancing the role of nurses in the technology development process, the importance of involvement extends to other user groups as well, such as patients.

In Sect. "Case Study: Developing a AI-based Continuous Monitoring Camera", we provide an overview of the case study. Subsection "Methods" details the methods used to conduct interviews with nurses. Section "Results" presents the results from these interviews. In Sect. "Discussion", we discuss the results and the case study in depth to address the research questions. Finally, in Sect. "Conclusion and Limitations", we discuss the limitations of this research and present our conclusions.

## Case Study: Developing a AI-based Continuous Monitoring Camera

In the Dutch FORSEE research project (Video monitoring FOR early Signalling of adverse EvEnts), the use of AI-based continuous video monitoring to early detect patients’ clinical deterioration is investigated. The project is carried out by the Eindhoven MedTech Innovation Center (e/MTIC), a collaboration between the Catharina Hospital, Eindhoven University of Technology and Royal Philips Eindhoven. Fontys University of Applied Sciences is also involved in the project. The research team is developing a monitoring camera, which is being tested at the cardiothoracic surgery department, which mainly houses patients after open heart surgery.

In hospitals, 40% of unexpected deaths occur in regular nursing wards (de Vries et al., 2008). Research has shown that mortality after surgical complications is more associated with hospital characteristics than with specific patient characteristics (Ghaferi et al., 2009). This means that hospital staff is not always equipped to take the necessary steps to timely recognize and treat complications in patients who reside in a nursing ward after surgery, in some cases resulting in the patients’ death. The inability to prevent death after a complication occurs is referred to as ‘failure-to-rescue’.

A track-and-trigger system was developed in 1997 to enable early detection of clinical deterioration in nursing ward patients, allowing timely interventions to prevent escalation. This socio-technical system organizes interactions between healthcare providers and technology and is used in hospitals worldwide. It tracks abnormalities in vital parameters—such as heart rate, oxygen saturation, blood pressure, respiratory rate, temperature, and consciousness level—that can signal clinical deterioration hours in advance (Churpek et al., 2012). Then, an Early Warning Score (EWS) is calculated to detect deterioration based on these vital parameters. A high EWS is associated with an increased likelihood of death (Subbe et al., 2001).

An increased EWS triggers a series of pre-defined medical and organizational responses that escalate based on severity, including more frequent patient observation, treatment, intensive care unit (ICU) admission, or calling the Medical Emergency Team (MET). METs, typically composed of doctors and nurses from the ICU or emergency department, aim to treat complications promptly and prevent further deterioration.

Yet, current monitoring methods for measuring the EWS have not reduced failure-to-rescue (Ludikhuize et al., 2015). This is likely due to how vital parameters are measured, processed, and interpreted. For instance, in the cardiothoracic surgery ward in this case study, nurses measure vital parameters manually three times daily. These intermittent ‘spot-checks’ offer a limited view of the patient’s EWS score. Patient-specific trends over time would provide more timely detection of clinical deterioration (van der Stam et al., 2023). Additionally, measuring the EWS is labor-intensive for nurses (Eddahchouri et al., 2021). Thus, there is a need to improve current EWS measurement practices.

To meet this aim, continuous EWS monitoring applications are being developed worldwide (e.g., van der Stam et al., 2023). The monitoring camera developed in the current project is an example of such an application and aims to improve the existing EWS monitoring practice in the cardiothoracic surgery ward. This camera aims to continuously measure vital parameters in a physically non-invasive and non-labor-intensive manner, which are then combined in a predictive model—based on the Early Warning Score—to timely alert nurses in case a patient deteriorates.

**Fig. 1:**
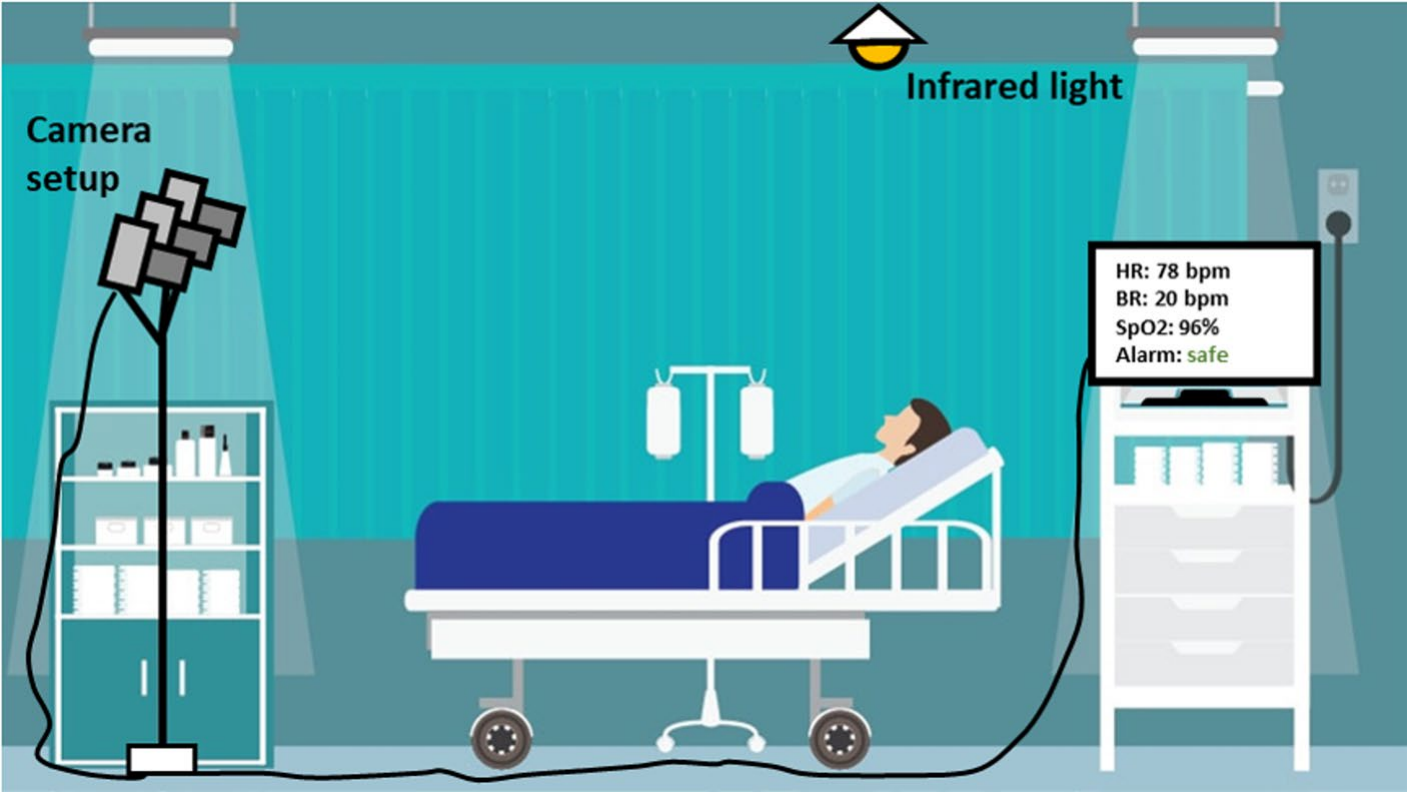
The set up of the FORSEE camera in the patients’ room

The camera is positioned in patient rooms to monitor the face and chest (see Fig. 1). It automatically measures heart rate by detecting color changes in the face due to pulsatile blood flow (Wang et al., 2017) and breathing frequency by detecting chest movements (Wang & den Brinker, 2022). Continuous monitoring creates patient-specific trends over time. Initial studies with other continuous monitoring sensors show positive effects on patient outcomes, such as a reduction in unplanned ICU admissions (Eddahchouri et al., 2022). Additionally, continuous monitoring would eliminate the need for nurses to manually measure the EWS, reducing their workload.

The FORSEE study followed a user-centered design approach guided by the Centre for eHealth Research (CeHRES) roadmap (van Gemert-Pijnen et al., 2011), which emphasizes iterative development, stakeholder involvement, and continuous evaluation in eHealth innovation. The research team actively involved both nurses and patients throughout the development and implementation of camera. The funding agency required the creation of a user committee, including nurses, to meet regularly and discuss the project’s progress. Collaboration with nurses was strengthened by including a research nurse, making the core team consist of a clinical researcher, a technical researcher, and a research nurse.

To ensure meaningful participation, the team regularly evaluated user experiences through questionnaires and information panels. Feedback from nurses directly influenced design decisions, such as the inclusion of a manual shutter on the camera to allow privacy when desired by either the nurse or the patient.

In addition to participatory design activities, the research team conducted several qualitative studies to explore how nurses and patients perceived the camera. The following data collection activities were conducted:

- Two focus groups with 20 nurses to discuss intended use, privacy, usability, and safety of the system.
- Two focus groups with 16 nurses to explore expectations regarding the system’s impact on personal contact and clinical reasoning.
- In-depth, semi-structured interviews with 8 patients, focusing on trust, safety, and privacy.
- In-depth, semi-structured interviews with 11 nurses to gather insights on their expectations regarding the impact of continuous video monitoring on socio-technical aspects of care, and their experiences and perspectives on being involved in the development of new healthcare technologies.

The outcomes of these focus groups and interviews are worked out in the FORSEE project. In that project, the studies contribute to the understanding of the value of personal interactions between nurses and patients, and to identify both facilitators and barriers to integrating remote monitoring technologies in clinical care.

In this paper, the 11 interviews with nurses are analyzed from a different perspective, as they have been conducted to investigate multiple research questions. These interviews have been conducted by the clinical researcher from the *FORSEE* project in collaboration with the Ethics department of the *Catharina Hospital* and two social scientists from the *Eindhoven University of Technology* and *the Rathenau Institute*. The findings presented here focus on how nurses perceive the impact of the camera on their work practices, how they experience their involvement in the development process of this technology, and how they believe they can be effectively engaged to shape the implementation of such technologies. Furthermore, this paper uses these insights to reflect on the broader implications of integrating AI-driven technologies in healthcare, and to offer lessons for ensuring that such innovations support human-centered care.

### Methods

Between October 2023 and March 2024, eleven semi-structured interviews were conducted with nurses working on the cardiothoracic ward where the camera was at that time being tested. The interviews lasted between 23 and 42 min, with a mean duration of approximately 30 min and a median duration of 30 min. To recruit interviewees, the researchers visited the nursing ward during a calm period of the day to find nurses available for an interview. Initially, all nurses who were available were interviewed, and later nurses were selected based on criteria such as age, years of experience, function, and gender, to ensure a mixed sample that is representative of the population at the ward.

The ages of the interview participants ranged from 21 to 56 years, with a mean age of approximately 28 years and a median age of 27 years. Participants had between 0.33 and 32 years of job experience, with a mean of approximately 7 years and a median of 5.5 years. The sample included 9 female and 2 male participants. In terms of professional roles, 5 were lead nurses, 5 were nurses, and 1 was a student nurse. The function ‘lead nurse’ refers to what in the Netherlands is known as ‘*regieverpleegkundige’.* The lead nurse performs both nursing and coordinating tasks within the ward.

The goal of the interviews was to collect nurses’ insights on two topics. The first is their expectation of how this camera will impact their ability to deliver care to patients, or, more briefly, the care practice. While most nurses were aware of the camera because it was being tested at the ward, it remains a challenge to interview future users about a technology that does not exist yet (Boenink et al., 2018). Therefore, the interviewers briefly explained the camera’s intended use and asked participants to reflect on how it might impact the care practice, both positively and negatively. The second topic is nurses’ experiences with and perspectives on being involved in technology development. The interview guide can be found in Appendix 1.

The interview analysis was structured as follows. First, all interviews were manually transcribed. These transcripts were then analysed using NVivo software, applying structural coding by assigning content-based or conceptual phrases to segments of data that relate to the research questions (Saldaña, 2021). Two researchers independently coded the transcripts.

A set of predefined codes was developed based on insights from prior focus groups and literature, focusing on themes such as the importance of personal contact between nurses and patients, and the impact of camera use on nurses’ clinical reasoning. The interview guide was tailored to elicit responses relevant to these topics, and transcripts were systematically screened for content aligned with these deductive codes.

In addition, the interviews included open-ended questions (e.g., “What do you like about your work?” and “How might the camera impact that?”), allowing for inductive analysis of emergent themes. These included aspects such as relationships with colleagues and patients, and overall job satisfaction.

Then, the initial codes were categorized in themes and refined iteratively. A frequency analysis was conducted to determine how often specific themes were mentioned, based on the number of individual participants referencing them. While the study is qualitative, this frequency data was considered relevant to the research goals. For example, findings such as “9 out of 10 nurses had no prior experience with being involved in technology development” provided meaningful context. The following section presents the themes that emerged from the interview data. These findings are used in Sect. Discussion  to answer the two research questions. The presented quotes are translated from Dutch for this article.

## Results

First, we present nurses’ expectations of the impact of this camera on the care practice. Second, we present nurses’ experiences with and perspectives on their involvement in technology development.

### Impact of the Camera on the Existing Care Practice

Nurses were mostly positive about the camera’s potential to earlier detect patient deterioration, and believe this could make their work more efficient, and might prevent acute situations. Overall, nurses were positive about the potential of the camera to support them in delivering care. For instance, a nurse said:

I think you can optimize human-to-human contact […] by adding supporting technologies (N1)

Many nurses emphasized the importance of feeling they provided ‘good care’ and did everything possible to help their patients. Nurses believed that the camera could contribute to delivering good care by the early detection of patient deterioration.

However, some nurses were wary about the camera’s potential to replace the care nurses deliver:

I do think it can help reduce the workload, but I hope they won’t use it as a full-time nurse (N2).

In line with that thought, nurses expressed that the camera could support them in their work, but could never “replace” them:

I don’t think there will ever be anything that can take over your clinical reasoning, your gut feeling, your communication, you have so many roles to take on as a nurse and monitoring is just one of them (N2)

To further understand what should not be replaced by the camera and why, the interviewers asked nurses to elaborate on which aspects of the current care routine should be maintained. The interviews revealed that the current practice of manually measuring the EWS three times daily ensures nurses have regular personal contact with patients. Currently, a patient’s EWS is measured using both manual and technology-assisted methods. Nurses enter the patient’s room three times a day with a cart of measuring devices, such as a digital ear-thermometer and a finger saturation meter. They record the vital parameters directly into the system, which automatically calculates and displays the EWS, then files it in the patient’s digital database. While the camera aims to replace this routine—partly to reduce the workload for nurses—the interviewed nurses actually value regular personal contact, and deem it a crucial element of the care practice. They believe personal contact is crucial for two reasons: patient well-being and for forming clinical judgment.

For the nurses, personal contact is crucial for patient well-being for social, psychological, and practical reasons. Firstly, patients have the need to socialize regularly. Without a nurse visiting, a patient might be alone at their room all day, making the presence of a nurse important, even if it is just for chatting. Secondly, patients can be in need of psychological support. Low spirits or anxiety about their condition can harm patients’ psychological well-being, potentially hindering their recovery. One nurse explains that

If someone has a negative attitude towards recovery, a patient will recover twice as slowly, and you won’t see that with figures, you will only notice that if you talk to someone about how things are going at that moment (N8)

Therefore, having a nurse around to offer a listening ear and to comfort the patient is essential. Thirdly, personal contact is essential for practical reasons, such as sharing information about the operation schedule and medication intake with the patient.

Further, personal contact is crucial for nurses to form clinical judgment. Nurses’ clinical judgment, or “clinical gestalt,” intuition, and gut-feeling are central to effective patient monitoring (Douw et al., 2015; Melin-Johansson et al., 2017). Clinical judgment is a reflective process based on practice, experience, knowledge, and critical analysis, leading to clinical conclusions (Cervellin et al., 2014; Connor et al., 2023), such as treatment decisions. Medical students and nurses learn to quickly assess a patient’s health status within seconds after entering the room (Cervellin et al., 2014). While vital parameters are important, they don’t provide the full picture. Nurses also rely on how patients look and interact to form their clinal judgment. Personal contact is essential for nurses to observe, feel, and communicate with patients to fully understand their condition. Additionally, when asked about their greatest source of job satisfaction, most nurses mentioned personal contact with patients.

In summary, the interviews revealed that manually measuring the EWS is a valuable socio-technical care practice. It ensures regular personal contact between the nurse and patient, which is vital for patient well-being, and allows the nurse to form clinical judgment, which is essential for effective monitoring. Additionally, personal contact is a key source of job satisfaction for many nurses.

Nurses worry that the camera could disrupt their routine of regular personal contact, as they would no longer need to visit patients three times a day to measure the EWS. While this could reduce their workload, some nurses feel it won’t make much difference due to other necessary visits, such as for wound care or patient requests. However, many fear that automated EWS measurements might lead to skipping visits to patients who don’t require specific care or don’t ask for help. If the automated measurements show a good score—especially during busy shifts—this could result in fewer patient visits and less personal contact, potentially undermining the care practice. Many nurses also worry that continuous monitoring may lead to over-reliance on automated measurements.

During the interviews, nurses shared personal experiences where automated measurements showed a good score, but their gut feeling indicated something was wrong, which was indeed the case. Therefore, they emphasized the importance of continuing to form clinical judgment and trust their gut feeling, even—or especially—with more measurements available. For instance, a nurse said:

Ultimately, you cannot promote care by deploying a robot. […] You really need feeling for that, judgment, and experience. So that is why I think it is very important that you continue with clinical reasoning (N8)

Nurses also stressed that learning to form clinical judgement should continue to be encouraged, especially among novice nurses. Finally, several nurses identified clinical reasoning as one of the most engaging aspects of their profession. One nurse expressed the worry that continuous monitoring might take over this task, making their profession increasingly focused on reading digits instead of “feeling and looking” (N8).

### Nurses’ Experiences with and Perspectives on Being Involved in Technology Development

The majority of nurses was interested in being involved in technology development. Some were interested in the technology itself and its technical specificities. Most said that being involved in technology development makes their work more engaging and diverse, allowing them to do something else besides their regular work.

While many nurses expressed interest in being involved in technology development, consulting nurses is not standard practice. Most nurses (9 out of 11) had not been involved in developing healthcare technology in their current or previous roles. One nurse noted that in other technology development projects, technology is typically implemented after completion. When nurses begin using the technology, it sometimes proves more time-consuming than expected. As a result, nurses may not use the technology due to time constraints, leading to a lack of results for researchers and jeopardizing the research. One nurse commented on this practice:

…we sometimes say: ‘this must have been invented by people who sit behind a desk’ (N10)

When the ‘added value’ of technology is unclear to nurses, using it can cause frustration, especially if it is time-consuming. ‘Added value’ here refers to technology improving measurable patient outcomes, such as shortening hospital stays or reducing complications. Many nurses shared negative experiences with a particular monitoring device currently used in their ward. They were dissatisfied with the tool because its added value was unclear, and using it is time-consuming. Additionally, nurses never received feedback from the developers about the technology’s impact on patient outcomes. Nurses said that

*…*when people feel that they have to do a lot, get little in return, see no results, are not kept informed, that starts to make people very irritated (N11)

and

*…*now we’ve just seen that it doesn’t actually help. Then it is no longer motivating, then no one wants to do it, but we have to keep doing it (N4)

The interviewers asked how this situation could be improved. The nurses’ answers can be summarized in two main recommendations. First, nurses believe their perspectives should be included early in the research project, because they feel their input is crucial for improving the practical workability of the technology. One nurse said that having a diverse array of perspectives on the care practice is important because

…[the developers] of course have a certain perspective on healthcare, but perhaps different. I think all those different perspectives will provide a very complete picture and therefore have a greater chance of success (N7)

Another nurse said:

It always sounds great behind the scenes, but you really need [nurses] to actually implement it in practice (N1)

Second, nurses emphasized that developers should share their findings to keep nurses informed about the technology’s added value:

…share results, show real examples, really discuss cases like wow, look at it, it has already yielded this result, so that people are really very closely involved in what they are doing (N11)

This will keep the nurses motivated to use the technology:

if you are a little further into the research and you found out that patients actually have a shorter hospital stay, then it is nice to hear that back because then you know: what we are doing makes sense (N5)

Some nurses mentioned their involvement in the* FORSEE *project as an example of good practice. Nurses believed that during this project, they were consulted at a sufficiently early stage during the development process, and their feedback was effectively implemented. A nurse said:

It’s not like: this is the technology, you’re going to work with it, and we hope it works. Now [the developers] are really looking at what we consider important and how it works in practice (N3)

Finally, all nurses believed that taking part in technology development falls under their tasks and responsibilities, because they deem it their responsibility to optimize care:

We are also trained to improve care and to look critically at the things we do, so I think we can and should think along with you in this regard (N5)

However, some nurses argued that the degree of involvement in such projects depends on individual factors such as personal interest. While some nurses are interested in the research aspect and collaborating with researchers, others prefer testing the technology in practice:

I’m more of a doer. I prefer to test it out and then provide feedback rather than actually researching what options are available and how we are going to approach it (N2)

## Discussion

In this section, we discuss the case study and interview results to answer the two research questions.

### The Results of Involving Nurses in the Development of an AI-driven Healthcare Technology

Here, we address this article’s first research question: What are the results of involving nurses in the development of AI-driven healthcare technology? The active involvement of nurses during the development of this technology, through the inclusion of a research nurse in the core research team and conducting focus groups and interviews with ward nurses, has yielded significant results for the further development and implementation of the camera, as well as the attitudes of the AI developers towards user involvement in the development process.

During the interviews, nurses acknowledged the camera’s potential to improve care practices by enabling early detection of patient deterioration and timely interventions. However, they also identified potential drawbacks, particularly the reduction in personal contact, which could negatively affect patient well-being, nurses’ clinical reasoning, and job satisfaction. These elements are currently fostered through regular personal contact between nurses and patients, making it an essential part of good care practice by expressing relevant care values (van Wynsberghe, 2013). If the camera replaces this practice and simply displays continuous measurements on a screen away from the patient, the valuable aspect of this care practice would be undermined.

The technology developers had not initially realized that measuring the EWS was such a valuable moment for personal contact, as the camera was designed to reduce nurses’ workload by replacing personal contact. Collaboration with nurses offered a new perspective: the camera’s development should not only ensure technical and clinical functionality but also be implemented in a way that preserves regular moments of personal contact. Nurses’ feedback emphasized that the project was not merely about creating functional AI technology, but about innovating a socio-technical care practice.

The current EWS measurement practice is already a highly mediated care practice, combining both social and technical elements, making it a socio-technical practice. By engaging with nurses and understanding their perspectives, developers realized that the camera must integrate positively into this existing care practice, preserving its core care values. They recognized that without nurses’ input on their experience and views of the care process and the role of technology, developing a technology that supports quality patient care would be impossible. As a result, the feedback led to key development requirements: the camera’s implementation should be paired with a new routine that ensures regular personal contact between nurses and patients, similar to the current practice of measuring EWS three times daily.

For example, continuous EWS measurements could be displayed on a portable device that nurses carry into patients’ rooms. This allows nurses to view measurements while with the patient, supporting their clinical reasoning. This could be integrated with other tasks, like wound care or medication administration. This way, the camera can enhance patient monitoring and reduce nurses’ workload while maintaining regular personal contact with patients, ensuring a good care practice.

It is well-documented in Science and Technology Studies and Philosophy of Technology literature that AI technology should be viewed as a socio-technical intervention. Scholars have shown that for a clinical intervention to be successful, AI applications must be considered socio-technical systems (Elish & Watkins, 2020; Smallman, 2022). Focusing solely on AI development is insufficient; the social and organizational context, relationships, and power dynamics surrounding AI implementation must be central to the research to ensure effective implementation (Elish & Watkins, 2020). Additionally, neglecting a socio-technical approach in designing new technology increases the risk of the system being deemed a ‘failure’ if it fails to provide the expected support to its users (Baxter & Sommerville, 2011).

This study reinforces these findings and highlights how involving nurses increased developers’ awareness of the value of user inclusion. It made developers realize that nurses’ perspectives are crucial not only during development but also in later implementation phases. Including the perspective of nurses postponed “the most obvious solution” (van der Velden & Mörtberg, 2015, p. 54) to solving the issue of failure-to-rescue, and allowed the researchers to explore alternative socio-technical scenarios with the camera in place. In this project, the development process was not understood as a collaboration between already formed groups, interests, and opinions, but rather as a process in which these groups, interests and opinions were formed (Binder et al., 2015; Santoni de Sio, 2024). This facilitated mutual learning (Rensing & Greenbaum, 2013; van der Velden & Mörtberg, 2015) as nurses gained insights into the issue and potential technological solutions, while researchers learned about the workplace, care activities, and nurses’ skills. Through collaboration, developers and nurses learned from each other’s perspectives and came to share each other’s stakes. This shows that user inclusion in practice can translate theoretical knowledge about the value of user involvement into tangible outcomes, fostering cohesion and potentially encouraging future user inclusion.

In conclusion, involving nurses in the development of an AI-driven healthcare technology can yield important insights for the development and implementation of the technology to preserve important care values, as well as steer the attitudes of AI developers towards understanding the value of user inclusion in the development process.

### The Significance of Involving Nurses in the Development of Healthcare Technology

Here, we address this article’s second research question: What is the significance of involving nurses in the development of AI-driven healthcare technology? It is crucial to assess the broader political and sociological effects of AI use in healthcare by considering long-term and diverse perspectives (Smallman, 2022). In the context of the nursing profession, the importance of involving nurses in healthcare innovation is particularly emphasized. Despite its evolution and progress over the past 60 years, structural issues persist, including low salaries, long working hours, and high levels of stress and burnout among nurses (Halter et al., 2017). These challenges contribute to a long-standing shortage of nurses in many countries (Drennan & Ross, 2019).

While automating care practices could help address staffing shortages, it might negatively affect the nursing profession. ‘Hands-on care’ is a crucial aspect of nursing (Wilson, 2002), and reducing personal contact could make the profession less appealing to both current and future nurses. This concern was echoed in interviews, where nurses expressed worries about automated measurements replacing personal contact with patients and other valued aspects of their job, such as clinical reasoning. Understanding healthcare digitization within this context highlights the need for nurses to have a role in shaping their profession. For example, Dutch government policy aims to address staffing shortages by promoting nurses’ autonomy and control over their work (Ministerie van Volksgezondheid, Welzijn en Sport, 2022). We propose that involving nurses in technology development is a key way to enhance their autonomy and control in the face of digitization, especially during significant staff shortages.

The interviews revealed that nurses are generally interested in being involved, both as co-developers and by testing technologies in practice. However, they noted that their involvement is not standard practice. Nurses reported using a device in their ward that they find impractical, non-functional, and time-consuming, leading to significant frustration. Additionally, researchers often fail to communicate clearly about the technology’s added value, which undermines nurses’ motivation to use and test it. Overall, nurses expressed that their input could greatly improve the practicality and effectiveness of new technology.

Therefore, the significance of involving nurses in the development of healthcare technology is twofold, impacting both the broader societal context and specific care contexts. In the broader societal context, promoting the autonomy and control of nurses in technology development could allow them to guide this development in a way that keeps their profession engaging amid digitization, which is particularly important given the current staffing shortages. And, empowering nurses to have control over the introduction of new technology that impacts their daily work can enable them to ensure its effectiveness and efficiency in a specific care context.

### Recommendations: Promoting Technological Citizenship in the Digitizing Health Care Domain

This study highlights the importance of recognizing the potential of users, such as nurses, and enabling their contribution to technology development. Citizens play various roles in shaping the impact of digitization on society, both in their private lives and in social settings like workplaces or research environments (Gardenier et al., 2024). However, institutional regulations, such as policies and norms within organizations, can limit their contributions if their potential is unrecognized. Nurses, who are typically not involved in technology development, exemplify this issue. As discussed in the interviews and reflected in the literature (Castner et al., 2016; Dykes & Chu, 2021; Hamer & Cipriano, 2013; Turan et al., 2017), nurses are generally excluded from technology development projects.

This suggests that the norms and rules within science and healthcare institutions typically do not support user involvement in the technology development process. Individuals within an organization, such as nurses and technology developers, have specific roles and are expected to behave accordingly (cf. Greif, 2006): developers focus on creating technically sound technology, while nurses provide bedside care. There are few, if any, established methods for these roles to interact, as there are no existing norms to facilitate collaboration.

In this case, the developers initiated collaboration with nurses, leading to mutual learning. Having experienced the benefits, the developers now aim to ensure future collaborations with nurses. This shift represents a change in the institutional system of science and healthcare, potentially establishing new norms around nurse involvement in technology development if these collaborations continue to succeed. For the nursing profession, this institutional change is especially significant. The profession has already undergone several waves of transformation. Traditionally, nurses were assistants to doctors, providing direct bedside care. Today, nurses can be specialists, and nursing care has advanced considerably. These changes were made possible by various institutional reforms (Bakker & aan de Stegge, 2020). Securing a role for nurses in healthcare technology development aligns with the ongoing evolution of the nursing profession.

To further promote the inclusion of nurses in (AI-driven) technology development, we offer several recommendations. Firstly, funding agencies should require the inclusion of nurses in technology development projects, especially when their practice is significantly affected by the technology being developed (see also Castner et al., 2016). Additionally, hospital managers could allocate more time and resources for nurses to participate in such projects (see also van Houwelingen et al., 2024). While this may seem counterintuitive given the current staffing shortage, involving nurses in research can actually save time and resources by preventing the development of ineffective technology. Based on the interviews, our recommendations include involving nurses at multiple stages of the research process, ensuring they are informed about technology outcomes to maintain motivation, and accommodating diverse contributions—whether research-focused or practice-oriented—to align with their varied interests. While this study focused on nurses, the importance of inclusion extends to other user groups, such as patients. Further research could explore how user involvement impacts technology development and fosters technological citizenship in healthcare.

## Conclusion and Limitations

This study demonstrated that involving users, particularly nurses, can enhance the innovation of a socio-technical care practice. It also emphasized the importance of user involvement for developers, translating insights from Science and Technology Studies and Philosophy of Technology into practice. However, the study had several limitations. First, nurses were interviewed during calm periods of their working hours to avoid using their free time. This scheduling constraint meant that not all nurses were available for interviews, limiting the number of participants. Additionally, the nurses who chose to participate were likely those with a particular interest in the technology and research process, potentially leading to a selection bias and excluding differing opinions. Despite these limitations, we believe the diversity in age, experience, background, and responses of the participating nurses provided a sufficiently broad range of views to achieve the aims of this study.

Ultimately, our findings suggest that including nurses in technology development enhances their agency in the ongoing digitization of their work, which is crucial for addressing the broader societal impact of AI in healthcare. Fostering technological citizenship in healthcare by empowering users and encouraging their participation can leverage AI’s benefits while mitigating risks, ultimately strengthening the resilience of healthcare systems in the digital age.

## Electronic Supplementary Material

Below is the link to the electronic supplementary material.

Appendix 1 Interview Guide

## Data Availability

The datasets generated and analyzed during this study, due to their sensitive and identifiable nature, as well as the restrictions imposed by the ethics protocol to safeguard the privacy of the subjects involved, are only available upon reasonable request.
