# Supplementary material for: Promoting User Involvement to Foster Technological Citizenship in the Digitizing Healthcare Domain
Source: Sci Eng Ethics. 2025 Nov 20;31(6):39. doi: 10.1007/s11948-025-00565-w (PMC12634806; doi:10.1007/s11948-025-00565-w)
Supplement: Supplementary file 1 — Appendix 1 Interview Guide [file 11948_2025_565_MOESM1_ESM.docx]

### **Appendix 1. Interview guide**

The semi-structured interviews were held in Dutch. For the purpose of this paper, the interview guide has been translated to English by the authors.

| INTRODUCTION |  |
| --- | --- |
| Goal of the research | Share with participant the goals of this research:   - Gathering insights on how this technology can effectively work in practice. - Reflecting on the involvement of nurses in this project |
| Introduction of the camera | Introduce the camera to the participant:   - Continuous automatic contactless Early Warning Score (EWS). - Aims: Preventing mortality and reducing workload - Feedback to nurse: yet unknown |
| Interview objectives | Share with participant the goals of this interview:   - Understanding how nurses perceive their profession - Understanding the potential impact of this technology on nursing - Understanding the experience and vision regarding involvement in technology development. |
| Personal information | Gather from participant the following information:   - Age - Years of work experience - Type of nurse |
| First impressions | - Ask participant about their first impressions of the smart camera |
|  | |
| Interview | |
| Themes  *“Themes” refer to previously defined codes from prior research.* | **TOPICS**  *The questions mentioned under “topics” are used as conversation starter with the participant, aimed to elicit responses relevant to the predefined codes. In addition, open-ended question are asked that aim to motivate the participant to discuss other topics as well.* |
| Impact of technology on nursing profession |  |
| Personal aspects | What do you enjoy about your work? |
|  | What gives you energy at work? |
|  | How might this camera influence these aspects? (postiviely or negatively) |
|  | What do you dislike about your work? |
|  | When have you had an unpleasant day? |
|  | How could this camera impact these aspects? (improve/worsen) |
|  | How do you believe the camera might impact your clinical judgement? |
|  | In case negatively: how could this be mitigated? |
| In relation to others  Doctor | How might the camera impact your relationship with the doctor? |
|  | - *E.g.: doctor is hard to call in when EWS measurements are good, but gut-feeling is not* |
|  | - *E.g.: EWS measurements give the nurse confidence to call the doctor in* |
| Patient | How do you value personal contact during the care practice (in case not already mentioned) |
|  | Why do you find personal contact important? (In case they deem it important) |
|  | When are you satisfied with the amount of personal contact you had with a patient? |
|  | When are you dissatisfied with the amount of personal contact you had with a patient? |
|  | How do you think the camera might influence this (amount/quality of personal contact)? |
| Role of nurses in developing new technology | Have you been involved in technology development projects before? If so, what was your experience? |
|  | Does contributing to technology development impact how your experience your work? |
|  | Do you believe this falls within your tasks and responsibilities? |
|  | Are you currently sufficiently supported to be involved in technology development projects? |
|  | What would you need to be involved in such research?   - *E.g.: working hours* |
|  | In what way could nurses be best involved in technology development projects?   - *E.g.: During which phase of the research, or in which role* |
